# Polarity‐Directed Synthesis of an Exfoliable 2D Polyoxometalate‐Based Metal‐Organic Framework for Noble Metal‐Free Alkyne Transfer Semi‐Hydrogenation

**DOI:** 10.1002/anie.7007153

**Published:** 2026-05-29

**Authors:** Xusheng Dai, Yalei Zhang, Yue Zhao, Shujun Li, Nana Ma, Qingchun Xia, Yiwei Liu, Rongji Liu, Shuxia Liu, Xuenian Chen, Carsten Streb

**Affiliations:** ^1^ Henan Key Laboratory of Boron Chemistry and Advanced Energy Materials Key Laboratory of Green Chemical Media and Reactions Ministry of Education, School of Chemistry and Chemical Engineering Henan Normal University Xinxiang China; ^2^ College of Chemistry and Materials Science Anhui Normal University Wuhu China; ^3^ Department of Chemistry Johannes Gutenberg University Mainz Mainz Germany; ^4^ Key Laboratory of Polyoxometalate and Reticular Material Chemistry of Ministry of Education College of Chemistry Northeast Normal University Changchun China

**Keywords:** 2D materials, hydrogenation, metal–organic frameworks, polyoxometalate, self‐assembly

## Abstract

Two‐dimensional polyoxometalate‐based metal–organic frameworks (2D POMOFs) hold great promise for catalysis, yet their directed synthesis and facile exfoliation remain challenging. Herein, we report the rational assembly of an exfoliable 2D POMOF, H_5_[(Co(H_2_O)_2_)_2_(C_18_H_12_N_6_)_4_(P_2_W_15_Ta_3_O_62_)]·18H_2_O (**1**), directed by the nonuniform charge distribution within the POM. The unique layered structure enables a multi‐step morphological control strategy: hydrothermal synthesis gives access to high‐quality single crystals, while a scalable bulk synthesis allows the rapid production of nanobelts, which can subsequently be exfoliated into ultrathin bilayer nanosheets via a liquid‐nitrogen treatment, or even monolayer nanosheets via solvent‐assisted sonication. The bilayer nanosheets exhibit high catalytic activity and selectivity for alkyne transfer semi‐hydrogenation, outperforming the corresponding bulk and nanocrystalline counterparts and rivaling noble‐metal‐based catalysts. This work establishes charge‐asymmetric POM‐directed synthesis as a viable strategy for creating defined 2D materials.

## Graphical abstract

Exfoliation of 2D nanosheets based on polyoxometalate metal‐organic frameworks (POMOFs) gives access to nanostructures with selective catalytic reactivity for the alkyne transfer semi‐hydrogenation which outperform the bulk references and rival some classical noble metal‐based catalysts.

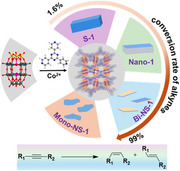

## 1 Introduction

The semi‐hydrogenation of alkynes is crucial for producing high‐purity olefins, yet it requires precise catalytic control to suppress over‐hydrogenation and double‐bond isomerization [1]. Direct hydrogenation using H_2_ is widely practiced but often requires high pressure and special equipment. Transfer hydrogenation using chemical hydrogen donors (e.g., formic acid, borohydrides, ammonia‐borane and alcohols) avoids pressurized H_2_, operates under mild conditions, and enables controlled hydrogen delivery to suppress over‐hydrogenation [2, 3, 4, 5]. These advantages have made transfer hydrogenation an important focus of hydrogenation research. However, reported catalysts for alkyne transfer hydrogenation are mainly homogeneous molecular complexes or heterogeneous metal nanoparticles [6, 7]. Although effective, these systems often suffer from poorly defined active sites, aggregation, or limited recyclability. Therefore, developing non‐noble metal heterogeneous catalysts with well‐defined structures, accessible active sites, and high durability is highly desirable.

Polyoxometalates (POMs) exhibit reversible redox activity and electron/proton reservoir capability, making them attractive as building blocks for catalytic hydrogenation [8, 9]. Some POMs can take advantage of their inherent Lewis acidity and redox properties to facilitate substrate activation and hydride transfer, and have therefore been applied in transfer hydrogenation of C═O bonds in biomass systems [10, 11]. However, the reported POM‐based systems typically need to be combined with noble‐metal single atoms or clusters (Pd, Pt, Au) for H_2_ activation and direct hydrogenation of unsaturated bonds (alkyne, alkene, nitroarene) [12, 13, 14, 15]. To the best of our knowledge, no non‐noble‐metal POM‐based catalyst for alkyne transfer semi‐hydrogenation has been reported to date.

Polyoxometalate‐based metal–organic frameworks (POMOFs) represent a class of hybrid materials that combine the merits of POMs with the high surface areas, tunable porosity, and structural periodicity of metal–organic frameworks (MOFs) [16, 17, 18, 19]. The incorporation of POMs as inorganic building blocks in POMOFs not only immobilizes and disperses these clusters, but also creates synergistic interactions between POMs, metal nodes, and organic ligands, thereby enhancing catalytic performance [20, 21]. Consequently, numerous POMOFs have been reported as efficient catalysts for acid‑promoted [22, 23], oxidative [24, 25], photocatalytic [26, 27, 28], and electrocatalytic [29, 30] reactions; however their potential for transfer hydrogenation remains largely unexplored.

Despite these advances, research has predominantly focused on three‐dimensional (3D) POMOFs, as conventional crystallization conditions tend to favor the formation of multi‐dimensional dense networks. However, the high structural stability of 3D frameworks often comes at a cost: the active sites, typically metal nodes and POM, can be embedded and obscured by organic ligands [31]. This significantly impedes mass transport and substrate accessibility, thereby limiting catalytic efficiency. Exfoliating layered POMOFs into two‐dimensional (2D) nanosheets presents a promising solution, as it exposes more active sites than 3D frameworks while preserving structural integrity [32, 33, 34]. Nevertheless, achieving high‑quality, large‑area 2D POMOFs remains challenging due to their inherent structural robustness, which often leads to fragmented, low‑aspect‑ratio materials upon conventional exfoliation. Therefore, innovative strategies for the controlled fabrication of well‐defined 2D POMOF nanosheets are urgently needed.

**SCHEME 1:**
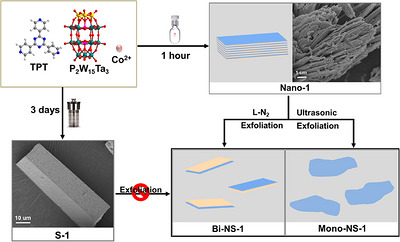
Schematic illustration of the morphological control strategy for the preparation of **S‐1**, **Nano‐1**, **Bi‐NS‐1,** and **Mono‐NS‐1**.

Herein, we present the directed assembly of an exfoliable 2D POMOF, H_5_[(Co(H_2_O)_2_)_2_(C_18_H_12_N_6_)_4_(P_2_W_15_Ta_3_O_62_)]•18H_2_O (**1**), guided by the nonuniform charge distribution of a Ta/W mixed‐addenda POM, K_5_Na_4_[P_2_W_15_(TaO_2_)_3_O_59_]•17H_2_O ({P_2_W_15_Ta_3_}), which acts as a structure‐directing agent. As illustrated in Scheme 1, a multi‐step morphological control strategy was developed for **1**: conventional hydrothermal synthesis of single crystals (**S‐1**) and a facile and rapid gram‐scale synthesis of nanobelts (**Nano‐1**). The resulting **Nano‐1** could be readily exfoliated into large‐area bilayer nanosheets (**Bi‐NS‐1**) via liquid nitrogen (L‐N_2_) treatment, as well as monolayer nanosheets (**Mono‐NS‐1**) by solvent‐assisted sonication. The obtained **Bi‐NS‐1**, with an aspect ratio of up to 5000:1, exhibits high activity and selectivity in the transfer semi‐hydrogenation of alkynes. To the best of our knowledge, **Bi‐NS‐1** represents the first example of a non‐precious‐metal POMOF catalyst for alkyne transfer semi‐hydrogenation.

## 2 Results and Discussion

**FIGURE 1:**
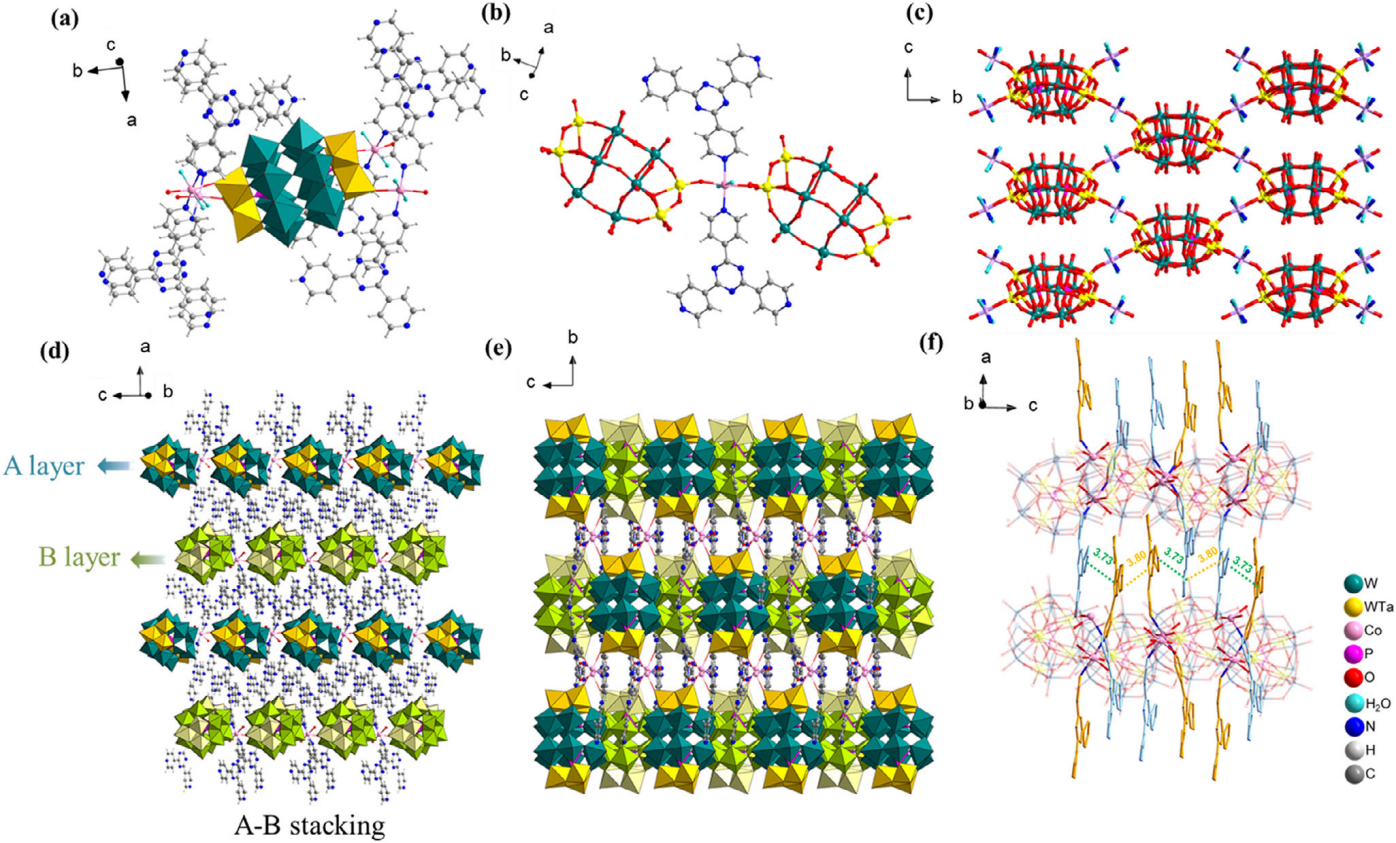
Crystal structure of **1**. (a) Coordination environment of {P_2_W_15_Ta_3_} connected to Co ions and TPT ligands (the coordinated water molecules are highlighted in cyan). (b) Local coordination geometry of the Co^2+^ ion. (c) The inorganic skeleton of single‐layer **1** (TPT ligands have been omitted for clarity). (d) Side view of the bilayer mixed polyhedral/ball‐and‐stick framework exhibiting A‐B‐type stacking. (e) Top view of the structure shown in (d), viewed along the *a‐axis*. *Note*: the distinct colors in 1d and 1e are used solely for visual clarity and do not indicate electronic or compositional differences. (f) Illustration of the interlayer stacking along the *a*‐axis, highlighting the π–π interactions.

Single‐crystal x‐ray diffraction analysis for **S‐1** (Table S1) demonstrates that **1** crystallizes in the orthorhombic system with space group *Aea2*. Its framework structure is directed by the Dawson‐type anion {P_2_W_15_Ta_3_}, which acts as the key inorganic building block. As shown in Figure 1a, the six polar metal sites of {P_2_W_15_Ta_3_} are statistically occupied by W and Ta atoms in a 1:1 ratio due to crystallographic orientational disorder. This disorder enables Co^2+^ coordination at both caps of the Dawson POM, thereby promoting their linkage into a 2D layered framework. The resulting structure features strong intralayer coordination bonds but weak interlayer interactions. This combination is key to the facile exfoliation of **Nano‐1** into large‐area nanosheets. Specifically, four of the six terminal O_t_(Ta/W) atoms serve as anchoring points, each coordinating to a {Co(TPT)_2_} complex (TPT = 2,4,6‐tri(pyridine‐4‐yl)‐1,3,5‐triazine, see Figure 1b). Each Co^2+^ center adopts a distorted octahedral geometry, ligated by two TPT ligands in a *trans* configuration, two O_t_(Ta/W) atoms from different {P_2_W_15_Ta_3_} clusters, and two aqua ligands (Figure 1b). Through these directional Co–O_t_ linkages, each {P_2_W_15_Ta_3_} unit connects to four neighboring counterparts, forming four (Ta/W)O_t–_Co–O_t_(Ta/W) bridges (Co–O bond lengths: 2.032–2.195 Å) that extend the structure into a well‐defined 2D single layer (Figure 1c).

Notably, each TPT ligand coordinates to a Co(II) ion through only one of its three pyridyl nitrogen atoms, leaving the remaining two nitrogen sites uncoordinated (Figure 1b). This monodentate coordination mode dictates the interlayer stacking. The 2D layers are stacked along the *a*‐axis in an A‐B stacking mode through weak interlayer π–π interactions (Figure 1d, Figure 1e and Figure S2b), with distances between adjacent TPT ligands ranging from 3.73 to 3.80 Å (Figure 1f). This stacking mode corresponds to a spatial offset arrangement, in which the A layer can be translated along the *c*‐axis by 6.68 Å to achieve complete overlap with the B layer. In the resulting architecture, two closely spaced TPT ligands from one layer intercalate into the space between two more distant ligands from the adjacent layer. This specific packing generates a distinctive “drawer‑like” interlocking structure (Figure S1c), which facilitates the subsequent exfoliation process.

The successful formation of this well‐defined layered architecture is attributed to the unique structure‐directing role of the charge‐asymmetric {P_2_W_15_Ta_3_} cluster. We have demonstrated in our previous work that the substitution of Ta^5+^ for W^6+^ not only increases the overall negative charge of the polyanion, but also induces a polarized charge distribution within the {P_2_W_15_Ta_3_} framework. The electron density at the O_t_(Ta) sites is significantly increased with high nucleophilicity, which enables them to preferentially coordinate with metal centers [35]. Control experiments using the Nb‐ or V‐substituted analogues {P_2_W_15_Nb_3_} or {P_2_W_15_V_3_} under identical conditions failed to yield an analogous framework, consistent with the lower nucleophilicity of their O_t_(Nb/V) groups [36]. These results underscore the critical role of {P_2_W_15_Ta_3_} in directing the assembly of this POMOF.

**Nano‐1** was synthesized in high yield via a scalable one‐pot protocol by heating the reactants in a sealed vessel at 150°C under stirring for 1 h, followed by cooling to room temperature (Scheme 1). Scanning electron microscopy (SEM) images reveal that **Nano‐1** adopts a belt‑like morphology, with widths ranging from 100 nm to 500 nm and lengths up to several micrometers (Figure S5). The IR spectra of **Nano‐1** and **S‐1** are completely identical (Figure S9). Furthermore, **Nano‐1** exhibits excellent chemical stability, retaining its structural integrity after 12 h of treatment in aqueous solutions across a broad pH range (pH 2 – pH 11) and in various organic solvents, including acetonitrile, ethanol, and *N,N*‐dimethyl formamide (Figure S10).

**FIGURE 2:**
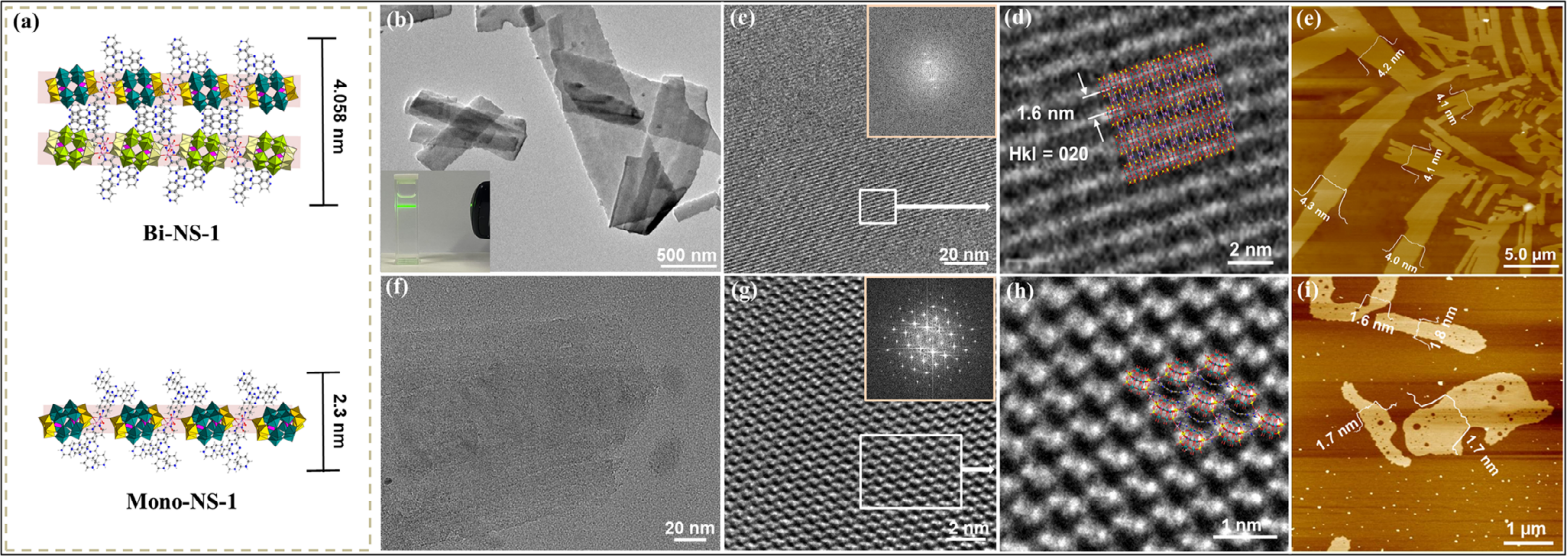
Structural characterization of bilayer Nanosheet (**Bi‐NS‐1**) and monolayer nanosheet (**Mono‐NS‐1**). (a) Schematic illustration of the structures and thicknesses of bilayer **Bi‐NS‐1** and monolayer **Mono‐NS‐1**. (b) TEM image of **Bi‐NS‐1** (inset: Tyndall effect of a **Bi‐NS‐1** suspension). (c) HRTEM image of **Bi‐NS‐1** and corresponding FFT patterns (inset). (d) Enlarged HRTEM image from the white solid lined square in part c, in which the bilayer single‐crystal structural model is embedded. (e) AFM image and corresponding height profile of **Bi‐NS‐1**. (f) TEM image of **Mono‐NS‐1**. (g) HRTEM image of **Mono‐NS‐1** and corresponding FFT patterns (inset). (h) Enlarged HRTEM image from the white solid lined square in part g, in which the monolayer single‐crystal structural model is embedded. (i) AFM image and corresponding height profile of **Mono‐NS‐1**.

Owing to the weak π–π stacking interactions between adjacent layers along the *a*‐axis (Figure 2a), **Nano‐1** could be readily exfoliated into nanosheets along the *bc* plane via two distinct routes (synthetic details are provided in the Supporting Information): liquid nitrogen (L‐N_2_) exfoliation yielded bilayer **Bi‐NS‐1**, while solvent‐assisted sonication in methanol produced monolayer **Mono‐NS‐1** (Scheme 1). However, the sonication route afforded **Mono‐NS‐1** only in low yield and with limited purity and the resulting monolayers showed a tendency to aggregate during collection by centrifugation. In contrast, the liquid‑nitrogen exfoliation provided **Bi‐NS‐1** with high yield, excellent purity, and high colloidal stability. Therefore, **Bi‐NS‐1** was selected as the primary material for the subsequent comprehensive characterization and catalytic studies, while **Mono‐NS‐1** was characterized only by electron microscopy.

Successful exfoliation of **Bi‐NS‐1** and **Mono‐NS‐1** was initially indicated by the Tyndall effect and confirmed by transmission electron microscopy (TEM) imaging (Figure 2b and f). For the bilayer **Bi‐NS‐1**, the high‐resolution TEM (HRTEM) images revealed distinct lattice fringes with a spacing of 1.6 nm (Figure 2c,d), matching the interlayer spacing of the (020) plane derived from the crystal structure. The selected‐area electron diffraction (SAED) patterns collected along the [100] axis (Figure S8b) exhibited diffraction spots assignable to the (020) and (002) planes, which align well with the simulated electron diffraction (ED) pattern of the bilayer structure along the same orientation (Figure S4b) and are consistent with the symmetry of the fast Fourier transform (FFT) analysis (Figure 2c). Atomic force microscopy (AFM) measurements show that the thickness of **Bi‐NS‐1** is predominantly distributed around 4.14 ± 0.16 nm (Figures 2e and S15), in good agreement with the theoretical bilayer thickness of 4.06 nm derived from the single‐crystal structure (Figure 2a).

For the monolayer **Mono‐NS‐1**, HRTEM images revealed a reticular (mesh‐like) structure (Figure 2f,g). The measured repeating unit of ∼0.85×0.5 nm corresponds well to the size of a single Dawson‐type POM cluster, and its in‑plane arrangement in the image is consistent with the single‐crystal structure of a monolayer (Figure 2h). The SAED patterns collected along the [100] axis showed spots attributed to the (020) and (011) planes (Figure S8d), which are in good agreement with the simulated single layer ED pattern (Figure S4d) and consistent with the symmetry of the corresponding FFT analysis (Figure 2g). AFM measurements indicated a thickness of ∼1.8 nm (Figure 2i), which is in line with the theoretical monolayer thickness of 2.3 nm derived from the single‐crystal structure (Figure 2a).

Finally, energy‐dispersive x‐ray spectroscopy (EDS) elemental mapping demonstrates the homogeneous distribution of Co, W, Ta, P, O, C, and N throughout **Bi‐NS‐1** (Figures S6 and S7).

**FIGURE 3:**
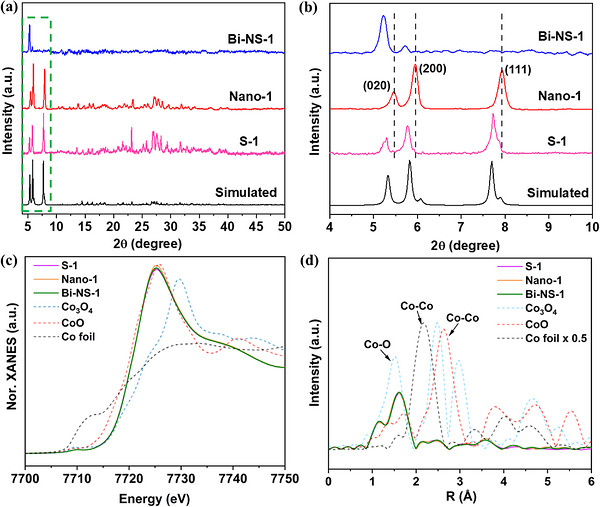
Comparative structural and spectroscopic analysis of **S‐1**, **Nano‐1,** and **Bi‐NS‐1**. (a and b) PXRD patterns, (c) Co *K*‐edge XANES, and (d) FT‐EXAFS spectra of Co_3_O_4_, CoO, and Co foil in the R space.

The phase purity, structural evolution, and local coordination environment of the cobalt sites throughout the morphological transformations were characterized by a combination of powder x‐ray diffraction (PXRD), x‐ray absorption spectroscopy (XAS), and Fourier transform infrared (FT‐IR) spectroscopy. As shown in Figure 3a, the PXRD patterns of both **Nano‐1** and **S‐1** closely match the simulated pattern of **S‐1**, confirming their high phase purity. Upon exfoliation from nanobelts (**Nano‐1**) to bilayer nanosheets (**Bi‐NS‐1**), distinct changes appear in the PXRD profiles (Figure 3b), including peak broadening, a pronounced preferred orientation of the (020) reflection, a marked attenuation of the (200) reflection, and the complete disappearance of the (111) reflection. The retention of a strong (020) peak alongside a diminished (200) peak indicates the loss of long‐range order along the stacking direction (*a*‐axis) while preserving in‐plane (*bc*‐plane) periodicity, which is consistent with successful exfoliation into ultrathin layers (see Figure S3 for the (200) and (020) planes). The electronic structure and coordination environment of Co were further probed by XAS. The Co X‐ray absorption near‐edge structure (XANES) spectra (Figure 3c) show that the absorption edge positions of **S‐1**, **Nano‐1,** and **Bi‐NS‐1** lie slightly higher than that of CoO, indicating that the Co centers remain in a cationic coordination environment. The Fourier‐transformed (FT) *k* ^3^‐weighted extended X‑ray absorption fine structure (EXAFS) spectra (Figure 3d) display a main peak at ∼1.6 Å, corresponding to the first‐shell Co–O/N coordination, with no detectable peak attributable to Co–Co scattering (∼2.2 Å). Finally, the FT‐IR spectroscopy confirms that the spectra of **S‐1**, **Nano‐1**, and **Bi‐NS‐1** are nearly identical (Figure S9), indicating that the exfoliation process does not alter the fundamental chemical bonds and functional groups within the framework. Taken together, these results indicate that the exfoliation process successfully yields ultrathin nanosheets while preserving the integrity of the local coordination environment and electronic structure of the Co active sites.

**FIGURE 4:**
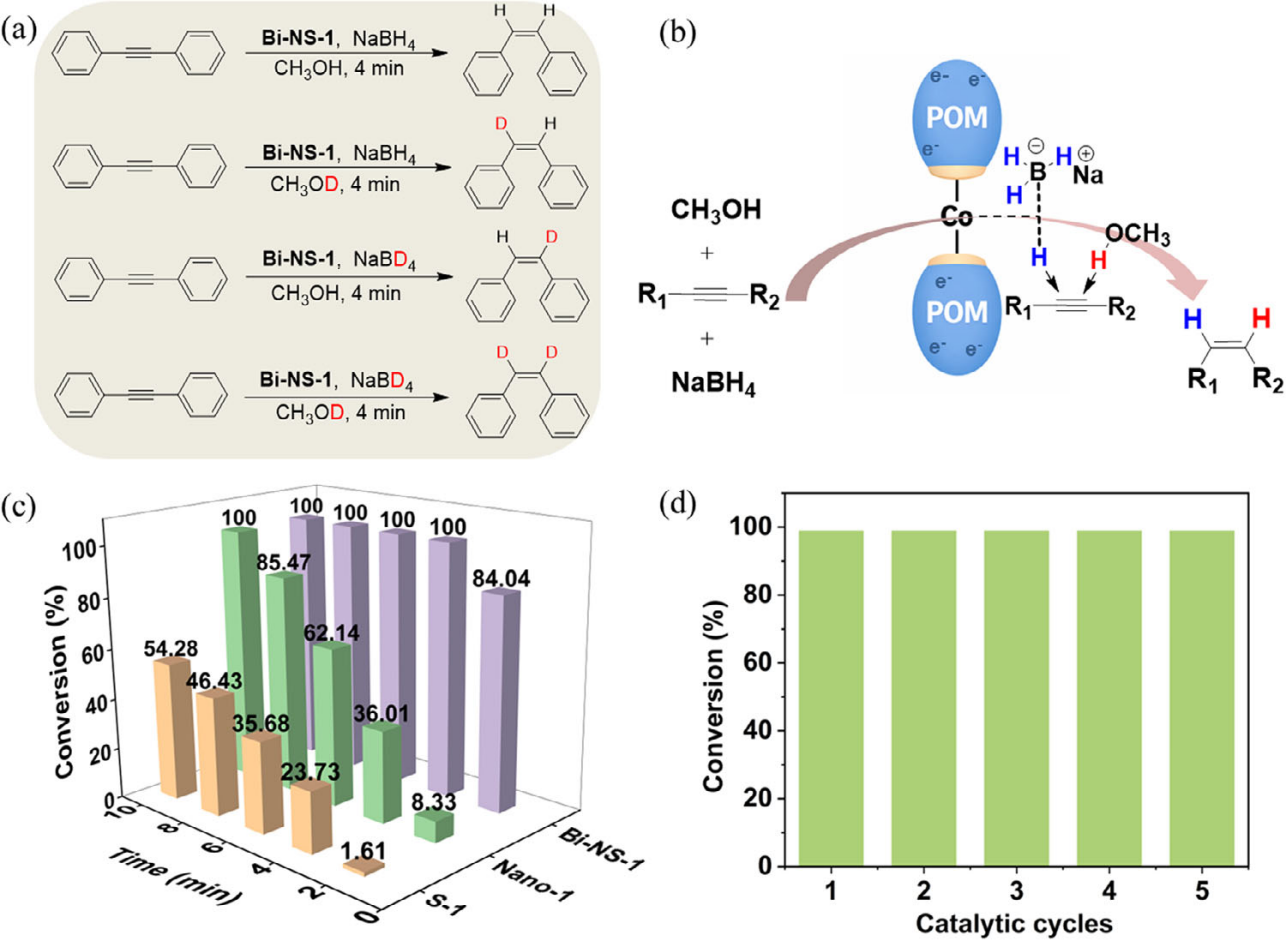
(a) Deuterium‐labeling experiments for the cis‐semihydrogenation of diphenylacetylene. (b) Proposed mechanism for the cis‐semihydrogenation of alkynes at the Co site. (c) Time‐dependent conversions for **S‐1, Nano‐1,** and **Bi‐NS‐1**. (d) Recyclability test of **Bi‐NS‐1**.

The catalytic performance of **1** was evaluated in the transfer semi‐hydrogenation of diphenylacetylene (DPA) using 2.5 equivalents of NaBH_4_ as the hydrogen source in methanol. As summarized in Table 1, **Bi‐NS‐1** achieved complete conversion of DPA within 4 min, with excellent selectivity, affording the alkene as the exclusive product without detectable over‑hydrogenation (Entry 1). In contrast, under identical conditions, **Nano‐1** and **S‐1** gave significantly lower conversions of 36% and 24%, respectively (Entries 2 and 3). Time‐dependent conversion profiles further highlight the superior activity of **Bi‐NS‐1**; for instance, with **S‐1** as catalyst, only 54% conversion was attained even after 10 min (Figure 4c). These results clearly demonstrate that the catalytic activity is highly dependent on the morphology of **1**, which directly influences the number of exposed active sites. Beyond a simple surface‐area effect, the distinct activity trend (**S‐1**< **Nano‐1**< **Bi‐NS‐1**) establishes a morphology‐dependent structure–performance relationship within the same POMOF framework. All three catalysts exhibited similarly high Z‑selectivity (Z/E ratios of 85:15, 90:10, and 86:14, respectively), indicating that the stereochemical outcome is an intrinsic property of the active sites within the layered framework of **1**.

**TABLE 1:** Control experiments for the transfer semi‐hydrogenation of diphenylacetylene.^a^

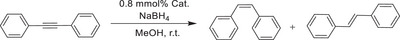				
			Selectivity (%)^b^	
Entry	Catalyst	Conv. (%)^b^	*Z*	*E*
1	**Bi‐NS‐1**	99	85	15
2	**Nano‐1**	36	90	10
3	**S‐1**	24	86	14
4	CoO^c^	<1	—	—
5	{P_2_W_15_Ta_3_}^d^	trace		
6	Co(NO_3_)_2_·6H_2_O + {P_2_W_15_Ta_3_}^e^	54	80	20
7	TPT^f^	trace		
8	Without catalyst	trace		
9	Without NaBH_4_	trace		

^a^ Reaction condition: substrate (0.2 mmol), catalyst (1.6 µmol), NaBH_4_ (0.5 mmol), Methanol (3 mL), room temperature; reaction time: 4 min.

^b^ Conversion was determined by GC‐MS with naphthalene as an internal standard.

^c^ 0.6 mg (2.75 µmol Co).

^d^ 1.2 µmol K_5_Na_4_[P_2_W_15_(TaO_2_)_3_O_59_]•17H_2_O.

^e^ physical mixture of 2.75 µmol Co(NO_3_)_2_·6H_2_O with 1.2 µmol K_5_Na_4_[P_2_W_15_(TaO_2_)_3_O_59_]•17H_2_O.

^f^ 0.4 µmol TPT ligand.

Control experiments show that CoO is nearly inactive under the standard conditions (<1% conversion, Entry 4), likely because its rigid six‐coordinated structure lacks accessible active sites. Using Co(NO_3_)_2_·6H_2_O alone as the catalyst led to the reduction of Co^2+^ to metallic Co^0^ nanoparticles. Neither the POM precursor {P_2_W_15_Ta_3_} nor the free ligand TPT exhibited appreciable catalytic activity (Entries 5 and 7). Moreover, a physical mixture of the Co^2+^ salt and {P_2_W_15_Ta_3_} initially exhibits catalytic activity; however, the formation of Co^0^ nanoparticles during the reaction leads to deactivation upon subsequent reuse (Entry 6). These observations indicate that neither the cobalt source alone nor the POM component alone is sufficient to account for the observed catalytic behavior.

During catalysis, **Bi‐NS‐1**, **Nano‐1,** and **S‐1** all turned deep blue, indicating the formation of reduced W‐containing species upon reduction by NaBH_4_. XPS analysis shows the emergence of W^5+^ species, while Co remained in the +2‐oxidation state (Figure S11c). In contrast, when the POM precursor {P_2_W_15_Ta_3_} was used alone as catalyst, no color change was observed and the XPS spectrum showed no evidence of tungsten reduction. These results are consistent with Co sites being responsible for NaBH_4_ activation, whereas the POM framework in **1** primarily acts as an electron reservoir.

Further evidence for electronic communication between the Co centers and the POM framework comes from XPS analysis. Compared with the POM precursor {P_2_W_15_Ta_3_}, the W 4f signals of pristine **S‐1** shift to lower binding energy (Figure S11a), whereas the Co 2p signal of **S‐1** shifts to higher binding energy relative to CoO (Figure S11b), suggesting partial electron transfer from Co to the POM through the Co–O–Ta/W connectivity. Upon treatment with NaBH_4_, a new W^5+^ signal appears together with the characteristic deep‐blue coloration (Figure S11c), indicating that the POM unit can accept electrons under reducing conditions. After subsequent addition of the alkyne, the W 4f peaks shift back to higher binding energy, while the Co 2p peaks shift to lower binding energy (Figure S11d), suggesting reversible electron redistribution between the POM framework and the Co centers during catalysis.

Taken together, these observations support a cooperative picture in which the Co sites and the POM framework play complementary roles during catalysis. In this cooperative process, the Co sites serve as the primary catalytic centers for borohydride activation, while the coordinated POM clusters stabilize the single‐ion Co sites both structurally and electronically. This buffering effect may help suppress over‐reduction and aggregation of cobalt species, thereby contributing to catalyst stability and sustained catalytic turnover.

To elucidate the hydrogen source in the alkene product, deuterium‐labeling experiments were performed. As shown in Figures 4a and S16–S19, when the reaction was conducted in CH_3_OD by using NaBH_4_ as the reductant, monodeuterated stilbene was obtained. Similarly, using NaBD_4_ as the reductant in CH_3_OH also afforded monodeuterated stilbene. In contrast, when both NaBD_4_ and CH_3_OD were used, dideuterated stilbene was isolated. These results demonstrate that the hydrogen atoms incorporated into alkene originate from the B–H bond of borohydride (as hydride, H^−^) and the O–H bond of methanol (as proton, H^+^). Based on these results, a mechanism is proposed as follows (Figure 4b): NaBH_4_ is activated at the Co center to generate a hydride species, which attacks the alkyne substrate. Concurrently, a proton is delivered from the hydroxyl group of methanol, enabling the formation of the alkene product. The POM cluster serves as a redox‐active electron reservoir that dynamically regulates the electronic state of Co centers, preventing their over‐reduction while sustaining catalytic turnover. Thus, electronic cooperativity within the Co‐POM unit enables efficient hydrogenation under mild conditions.

With the optimized reaction conditions established, the substrate scope of **Bi‐NS‐1** was investigated using a range of internal alkynes. As shown in Table S4, various para‐substituted diphenylacetylenes bearing either electron‐withdrawing (halogen) or electron‐donating groups (methyl, ethyl, methoxy) were efficiently transformed into the corresponding alkenes with high conversions (99%) and selectivity for the alkene (99%). The minimal influence of substituent electronic properties on the reaction outcome underscores the broad applicability of **Bi‐NS‐1**.

The stability and reusability of **Bi‐NS‐1** were also evaluated. After the reaction, **Bi‐NS‐1** was recovered by centrifugation, washed with methanol and dried under vacuum before direct reuse. As shown in Figure 4d, **Bi‐NS‐1** retained high catalytic activity over five consecutive cycles with only a negligible decrease in yield. The recycled catalyst gradually recovered from blue to its original orange‐yellow color after exposure to air for one hour. PXRD and FTIR analyses of the recovered **Bi‐NS‐1** confirmed that the crystalline structure remained intact (Figure S12). Moreover, XPS data indicated that the oxidation state of W^6+^ and Co^2+^ were preserved after the catalytic reaction (Figure S13), further affirming the robust stability of the catalyst under operational conditions.

## 3 Conclusion

In summary, this work demonstrates a charge‑asymmetric POM‑directed strategy for the rational construction of 2D crystalline POMOFs. By exploiting the uneven charge distribution of the mixed‐addendum cluster {P_2_W_15_Ta_3_}, we successfully synthesized a robust framework featuring a unique “drawer‐like” interlayer structure. This specific architecture enabled a precise morphological control, leading to the scalable preparation of distinct materials: from bulk single crystals (**S‐1**) to nanobelts (**Nano‑1**), and finally to bilayer nanosheets (**Bi‐NS‐1**), and monolayer nanosheets (**Mono‐NS‐1**). The ultrathin **Bi‐NS‐1** exhibits high activity in the transfer semi‑hydrogenation of alkynes at room temperature. Mechanistic studies support a cooperative picture involving the Co^2+^ sites and the POM clusters. Co centers activate hydride from NaBH_4_, while the POM clusters provide structural support, stabilize Co^2+^ centers, and serve as an electron reservoir that buffers local charge density, which may help suppress over‑reduction and aggregation of cobalt species. Notably, **Bi‐NS‐1** also maintains high selectivity and robust stability over multiple cycles. Collectively, this study establishes a feasible synthetic strategy in which charge‑asymmetric POMs dictate the assembly and morphological evolution, providing a versatile route to functional 2D POMOF materials.

## Author Contributions

**Xusheng Dai**: investigation, methodology, formal analysis, visualization, writing – original draft, data curation, writing – review and editing. **Yalei Zhang**: investigation, methodology, writing – original draft. **Yue Zhao**: investigation, formal analysis, writing – review and editing. **Shujun Li**: conceptualization, funding acquisition, writing – review and editing, writing – original draft, formal analysis, validation, project administration, resources, supervision. **Nana Ma**: conceptualization, methodology, formal analysis, investigation, writing – review and editing. **Qingchun Xia**: investigation, validation, formal analysis. **Yiwei Liu**: conceptualization, investigation, methodology, formal analysis, writing – review and editing, writing – original draft, visualization. **Rongji Liu**: methodology, investigation, formal analysis, data curation, writing – review and editing. **Shuxia Liu**: investigation, formal analysis, writing – review and editing. **Xuenian Chen**: supervision, resources, formal analysis, writing – review and editing. **Carsten Streb**: conceptualization, supervision, resources, project administration, funding acquisition, writing – review and editing, validation.

## Conflicts of Interest

The authors declare no conflicts of interest.

## Supporting information




**Supporting file 1**: anie72937‐sup‐0001‐SuppMat.pdf.
Synthetic, experimental and catalytic data are reported in the Supporting Information. CCDC 2375071 contains the supplementary crystallographic data for this paper. These data can be obtained free of charge from The Cambridge Crystallographic Data Centre via “www.ccdc.cam.ac.uk/structures” The authors have cited additional references within the Supporting Information [37, 38, 39, 40, 41, 42, 43, 44].

**Supporting File 2**: anie72937‐sup‐0001‐Data.zip.

## Data Availability

The data that support the findings of this study are available from the corresponding author upon reasonable request.
