# Supplementary material for: Polarity‐Directed Synthesis of an Exfoliable 2D Polyoxometalate‐Based Metal‐Organic Framework for Noble Metal‐Free Alkyne Transfer Semi‐Hydrogenation
Source: Angew Chem Int Ed Engl. 2026 May 29;65(29):e7007153. doi: 10.1002/anie.7007153 (PMC13360626; doi:10.1002/anie.7007153)
Supplement: Supplementary file 1 — Supporting file 1: anie72937‐sup‐0001‐SuppMat.pdf. Synthetic, experimental and catalytic data are reported in the Supporting Information. CCDC 2375071 contains the supplementary crystallographic data for this paper. These data can be obtained free of charge from The Cambridge Crystallographic Data Centre via “www.ccdc.cam.ac.uk/structures” The authors have cited additional references within the Supporting Information [37, 38, 39, 40, 41, 42, 43, 44]. [file ANIE-65-e7007153-s002.pdf]

Supporting Information

Polarity-Directed Synthesis of an Exfoliable 2D Polyoxometalate-Based Metal-Organic Framework for Noble Metal-Free Alkyne Transfer Semi-Hydrogenation

Xusheng Dai,[a] Yalei Zhang,[a] Yue Zhao,[a] Shujun Li,*[a] Nana Ma,[a] Qingchun Xia,[a] Yiwei Liu,*[b] Rongji Liu,[c] Shuxia Liu,[d] Xuenian Chen,[a] Carsten Streb*[c]

[a] X. Dai, Y. Zhang, Y. Zhao, S. Li, N. Ma, Q. Xia, X. Chen,
Henan Key Laboratory of Boron Chemistry and Advanced Energy Materials
Key Laboratory of Green Chemical Media and Reactions, Ministry of Education
School of Chemistry and Chemical Engineering, Henan Normal University
Xinxiang, 453007, China
E-mail: lisj@htu.edu.cn
[b] Y. Liu
College of Chemistry and Materials Science, Anhui Normal University
Wuhu, 241002, China
E-mail: liuyw@ahnu.edu.cn
[c] R. Liu, C. Streb
Department of Chemistry, Johannes Gutenberg University Mainz,
Duesbergweg 10–14, 55128 Mainz, Germany
E-mail: carsten.streb@uni-mainz.de
[d] S. Liu
Key Laboratory of Polyoxometalate and Reticular Material Chemistry of Ministry of Education
College of Chemistry, Northeast Normal University
Changchun, 130024, China

S1 Experimental procedures

1.1 Materials and Instruments

The precursor $K_5Na_4[P_2W_{15}(TaO_2)_3O_{59}]\cdot 17H_2O$ was synthesized according to the procedure described in our previous report.[37] 2,4,6-Tri-4-pyridinyl-1,3,5-triazine (TPT), 97% (Aladdin); Cobaltous nitrate hexahydrate ($Co(NO_3)_2\cdot 6H_2O$), 99% (Aladdin); Methanol (MeOH), AR, 99.8% (Macklin); 1,2-Bis(4-methoxyphenyl)ethyne, 97% (Macklin); 1,2-Di-p-tolylethyne, 97% (Bidepharm); 1-Ethyl-4-[2-(4-Ethylphenyl)Ethynyl]Benzene, 96% (Aladdin); 1,2-Bis(4-fluorophenyl)ethyne, 97% (Macklin); Diphenylacetylene, 99% (Macklin); Sodium bisulfite, 97% (Macklin); Sodium hydroxide (NaOH), AR, 96% (Aladdin); Chloroform-d (CCl_3D), 99.8%D, contains 0.03 %(v/v) TMS (Energy Chemical); Dimethyl sulfoxide-d6 (DMSO-d6), 99.8%D, contains 0.03 %(v/v) TMS (Energy Chemical). Deionized water is used during the whole experiments. All the other reagents were obtained commercially and used without further purification.

FT-IR analysis in ATR mode was performed by a Perkin Elmer Spectrum 400 FT-IR/FT-FIR Spectrometer equipped with ATR module in the range of 400−4000 cm^{-1} at room temperature.

Powder X-ray diffraction (PXRD) measurements were performed on a Panalytical X'Pert3 Powder diffractometer with graphite monochromatized Cu *Kα* radiation (λ = 0.1541 nm, 40 KV, 150 mA) at 298 K.

Elemental analyses for Co, P, Ta and W were determined with a Agilent 7800(MS) ICP atomic emission spectrometer. Elemental analysis for C was performed on a Vario EL cube elemental analyzer.

Thermal analyses were performed on a Netzsch 449C thermal analyzer. The sample was heated to 1000 °C with a heating rate of 10 °C/min, under an N_2 atmosphere.

Scanning electron microscope (SEM) analysis was conducted on a ZEISS GeminiSEM 300 scanning electron microscope equipped.

High-resolution transmission electron microscopy (HRTEM) analysis was conducted on a JEM-F200 transmission electron microscope equipped with a super energy-dispersive spectrometer (EDS). TEM samples were prepared by casting several drops of a sample solution onto copper-mesh TEM grid mounted with a holey carbon film.

X-ray photoelectron spectroscopy (XPS) measurements were performed on Thermo Fisher Scientific ESCALAB250Xi X-ray photoelectron spectroscopy.

Atomic Force Microscopy (AFM) The thickness of the **Bi-NS-1** and **Mono-NS-1** were gained in a tapping model by atomic force microscope (AFM) instrument (Bruker Corporation, UK) controlled with the Nanoscope V software V8.

X-ray absorption fine structure (XAFS) spectra of Co were acquired at BL11B station in Shanghai Synchrotron Radiation Facility (SSRF, China), operated at 3.5 GeV with a maximum current of 250 mA and using a Si (111) double-crystal monochromator. The XAFS data were collected under ambient conditions in fluorescence mode using a Lytle detector. Co foil, CoO and Co_3O_4 were used as reference samples.

1.2 Synthesis

Synthesis of S-1

Samples of $K_5Na_4[P_2W_{15}(TaO_2)_3O_{59}]\cdot 17H_2O$ (0.2 g, 0.04 mmol) and solid $NaHSO_3$ (0.04 g, 0.38 mmol) were added in 10 mL of 80 °C deionized water and stirred until the yellow solution became colorless. Then, $Co(NO_3)_2\cdot 6H_2O$ (0.0291 g, 0.1 mmol), 2,4,6-tri(Pyridin-4-yl)-1,3,5-triazine (0.0375 g, 0.12 mmol) were added into the resulting solution, respectively. After adding 5 mL methanol, 1 M sodium hydroxide was added to adjust the pH value of the mixed solution to 7.0 and stirred at room temperature for another 10 min. The suspension was transferred into a 25 mL Teflon-lined autoclave and then heated for 3 days under autogenous pressure at 160 °C. After cooling down to room temperature at a rate of 10 °C/h, orange crystals were isolated and washed with distilled water and methanol, and then dried in air to obtain desired single-crystal product of **S-1** in a yield of 48 % (based on $K_5Na_4[P_2W_{15}(TaO_2)_3O_{59}]\cdot 17H_2O$). Anal. Calcd. (%): Co 1.91, P 1.00, Ta 8.82, W 44.80. Found: Co 1.20, P 1.15, Ta 9.24, W 45.31. IR (KBr disks): 1514 (m), 1372 (m), 1315 (w), 1087 (m), 1013 (w), 944 (m), 899 (m), 743 (vs), 663 (vw), 646 (vw), 595 (vw), 561(vw), 511 (s), 464 (vw) cm^{-1}.

Synthesis of Nano-1
Samples of $K_5Na_4[P_2W_{15}(TaO_2)_3O_{59}]\cdot 17H_2O$ (0.4 g, 0.08 mmol) and solid $NaHSO_3$ (0.08 g, 0.76 mmol) were added in 20 mL of 80 °C deionized water and stirred until the yellow solution became colorless. Then, $Co(NO_3)_2\cdot 6H_2O$ (0.0582 g, 0.2 mmol), 2,4,6-tri(Pyridin-4-yl)-1,3,5-triazine (0.075 g, 0.24 mmol) were added into the resulting solution, respectively. After adding 10 mL methanol, 1 M sodium hydroxide was added to adjust the pH value of the mixed solution to 7.0 and stirred at room temperature for another 10 min. The suspension was transferred into a 48 mL pressure-resistant bottle, heated and stirred at 150 °C under self-pressure for 1 h. After heating was stopped, the mixture was stirred until cooled to room temperature. The orange powder is separated, washed with distilled water and methanol, and then dried in air to obtain the required product of **Nano-1** with a yield of 51% (based on $K_5Na_4[P_2W_{15}(TaO_2)_3O_{59}]\cdot 17H_2O$).

Synthesis of Bi-NS-1
Nano-1 (5 mg) was placed in a 10 mL glass vial, and methanol (3 mL) was added. The vial was immersed in liquid nitrogen to completely freeze the solution and then rapidly transferred to an 80 °C water bath to thaw. This freeze–thaw cycle was repeated six times. The vial was subsequently kept at room temperature for 15 min. Finally, the suspension was centrifuged to collect the precipitate, which was obtained as **Bi-NS-1** and dried under vacuum at room temperature, giving an isolated yield of ca. 40 wt %.

Synthesis of Mono-NS-1
Freshly prepared **Nano-1** (10 mg) was dispersed in methanol (10 mL) and subjected to ultrasonic treatment at room temperature using a 100 W ultrasonic bath for 30 min. The resulting suspension was then centrifuged at 800 rpm for 3 min to remove non-exfoliated microcrystals, followed by further centrifugation at 3000 rpm for 5 min to collect the precipitate as **Mono-NS-1**.

S2 X-ray crystallography

Single crystal XRD (SCXRD) analysis of **S-1** was recorded on an Agilent SuperNova Dual diffractometer using graphite-monochromated Cu Kα radiation, λ = 1.54184 Å. The linear absorption coefficients, scattering factors for the atoms, and anomalous dispersion corrections were taken from the International Tables for X-Ray Crystallography. Empirical absorption corrections were applied. Structures were solved using direct methods (SHELXT)[38] and refined by full-matrix leastsquares (SHELXL) interfaced with the program OLEX2[39] Anisotropic thermal parameters were used to refine all non-hydrogen atoms, with the exception for a few oxygen atoms. Those hydrogen atoms attached to lattice water molecules were not located. The lattice water molecules were estimated by thermogravimetry and only partial oxygen atoms of water molecules were observed by X-ray structure analysis. The crystal data and structure refinement results are summarized in Table S1. Further details on the crystal structure investigation scan be obtained free of charge from The Cambridge Crystallographic Data Centre via www.ccdc.cam.ac.uk/data_request/cif on quoting the depository number CCDC 2375071 (**1**).

Table S1. Crystal data and structural refinements for **S-1**.

Compounds	**1**
Formula	$C_{72}H_{58}Co_2N_{24}O_{68}P_2Ta_3W_{15}$
Formula weight (g·mol^{-1})	5821.77
T (K)	170.00(10)
Wavelength (Å)	1.54184
Crystal	orthorhombic
Space group	Aea2
a (Å)	30.3133(5)
b (Å)	33.1363(6)
c (Å)	13.3688(3)
α (˚)	90
β (˚)	90
γ (˚)	90
V (Å^3)	13428.6(4)
Z	4
D_{calc} (mg m^{-3})	2.880
μ (mm^{-1})	30.290
F (000)	10436.0
Crystal size (mm^3)	0.02 × 0.013 × 0.01
Goodness-of-fit on F^2	1.048
Final *R* indices	R_1 = 0.0820
[I >2σ(I)][a]	wR_2 = 0.2218
R indices[a]	R_1 = 0.0901
(all data)	wR_2 = 0.2332
Flack parameter	0.17(3)

[a] $R_1 = \sum||F_o|-|F_c||/\sum|F_o|$; $wR_2 = \{\sum[w(F_o^2-F_c^2)^2]/\sum[w(F_o^2)^2]\}^{1/2}$

S3 Structural representations

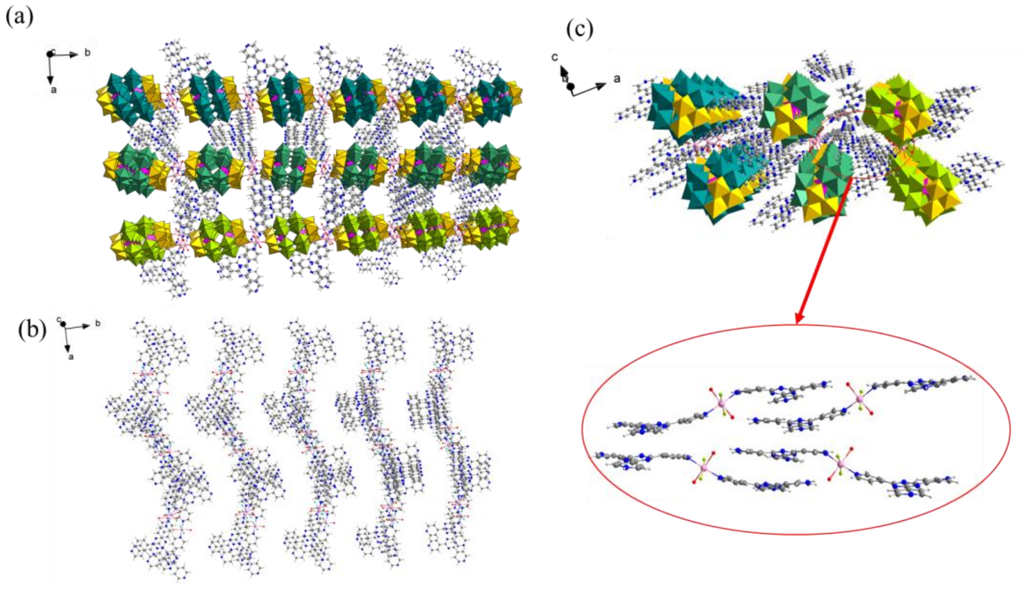

Figure S1. (a) Mixed polyhedral/ball-and-stick representations of the three layers of POMOF connected by π-π stacking interactions, (b) organic components, (c) local amplification of ligand stacking by π-π stacking interactions.

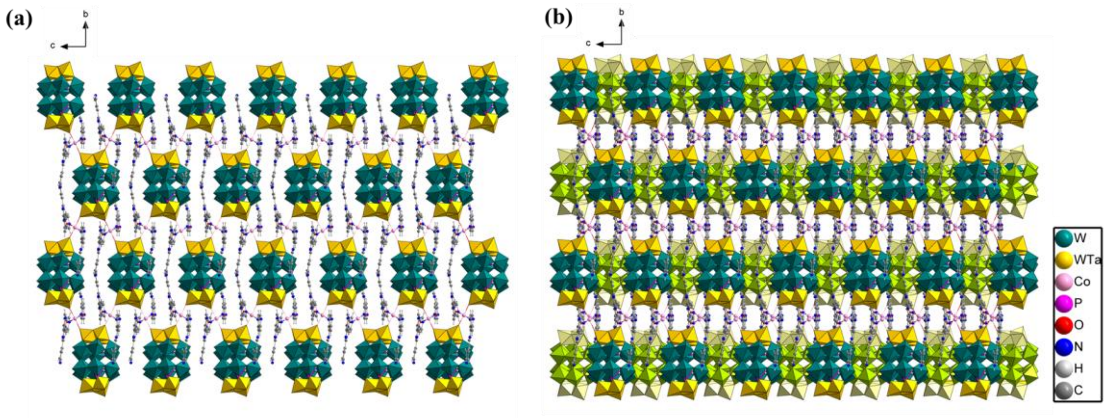

Figure S2. Mixed polyhedral/ball-and-stick representations of (a) the monolayer and (b) the bilayer structures of **1** along the [100] direction.

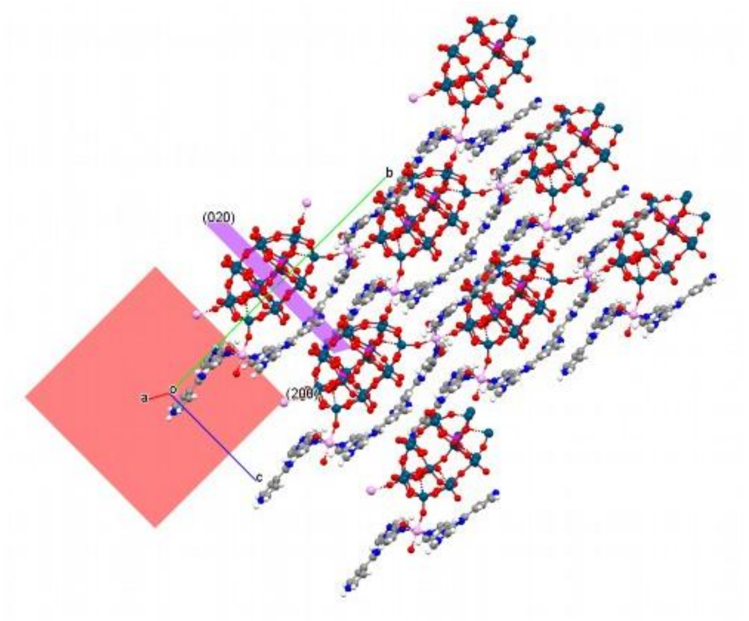

Figure S3. The structure of **1** highlighting the (200) and (020) planes by red and purple, respectively. Color code for atoms: W and Ta/W (light blue), Co (pink), P (purple), O (red), C (grey), N (blue), H (white).

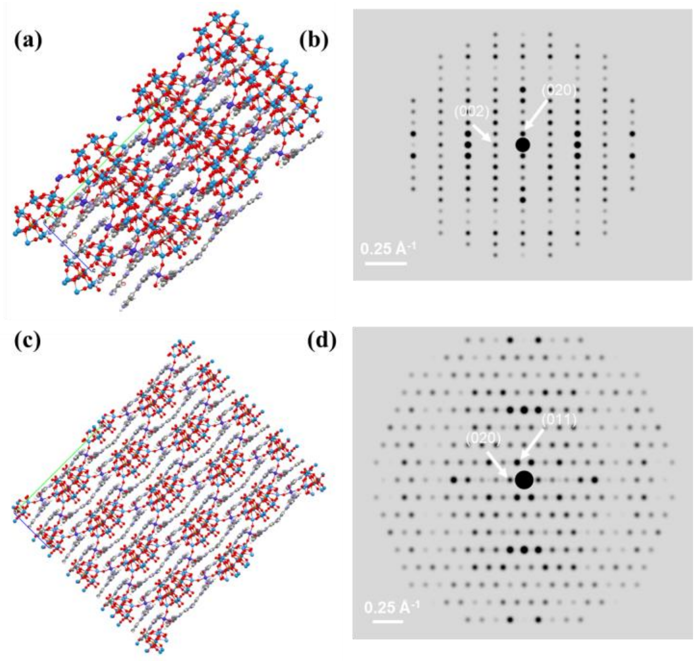

Figure S4. (a) The bilayer nanosheet along the [100] direction in the single-crystal structure of **1**. (b) Simulated electron diffraction (ED) pattern of bilayer nanosheet along the [100] direction. (c) The monolayer nanosheet along the [100] direction in the single-crystal structure of **1**. (d) Simulated electron diffraction (ED) pattern of monolayer nanosheet along the [100] direction.

The simulated ED pattern was produced by the Crystalmaker software (http://crystalmaker.com/) using the CIF file of the parent bilayer and monolayer single-crystal structure.

Table S2. Bond valence calculations of select O atoms and N atoms of **1**.

Atom	Bond	Distance / Å	Bond Valence	Bond Valence Sum (BVS)
O1	O1-Co1	2.070	0.360	0.360
O2	O2-Co1	2.101	0.331	0.331
O3	O3-Co1	2.123	0.312	1.895
O3	O3-Ta1	1.750	1.583	
O4	O4-Co1	2.124	0.311	1.795
O4	O4-Ta2	1.774	1.484	
N1	N1-Co1	2.127	0.460	2.91
N1	N1-C1	1.399	1.212	
N1	N1-C2	1.391	1.238	
N2	N2-Co1	2.162	0.419	3.029
N2	N2-C3	1.375	1.293	
N2	N2-C4	1.368	1.317	

Table S3. Bond valence sum calculations of Co ions of **1**.

Atom	Bond	Distance / Å	Bond Valence	Bond Valence Sum (BVS)
Co1	Co1-O1	2.070	0.360	2.193
	Co1-O2	2.101	0.331	
	Co1-O3	2.123	0.312	
	Co1-O4	2.124	0.311	
	Co1-N1	2.127	0.460	
	Co1-N2	2.162	0.419	

S4 Principal characterization

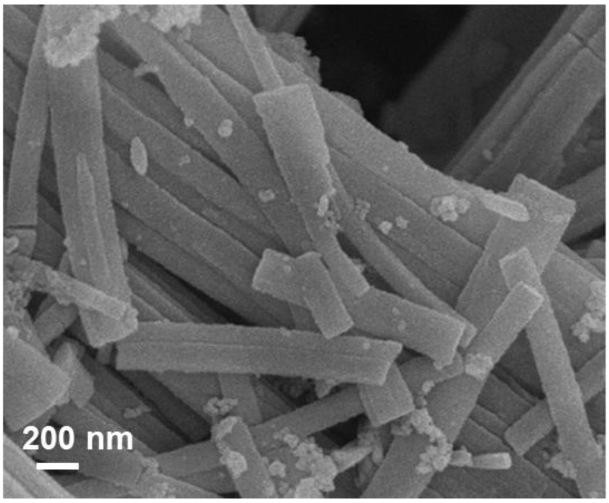

Figure S5. SEM image of **Nano-1**.

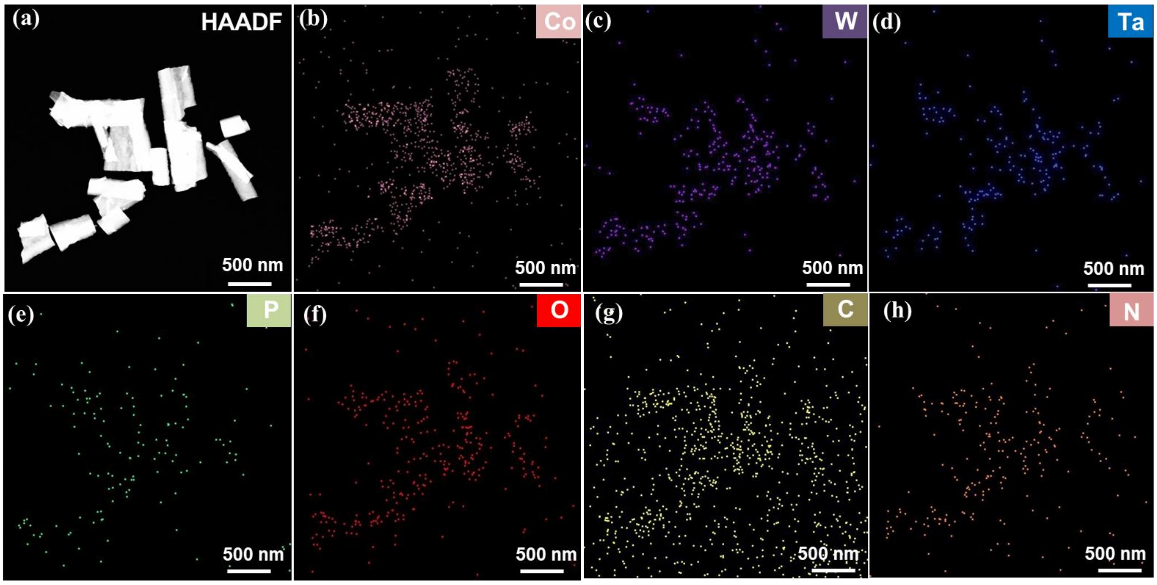

Figure S6. (a) HAADF-STEM image of **Bi-NS-1** and (b-h) TEM-EDX elemental mapping images of Co, W, Ta, P, O, C and N.

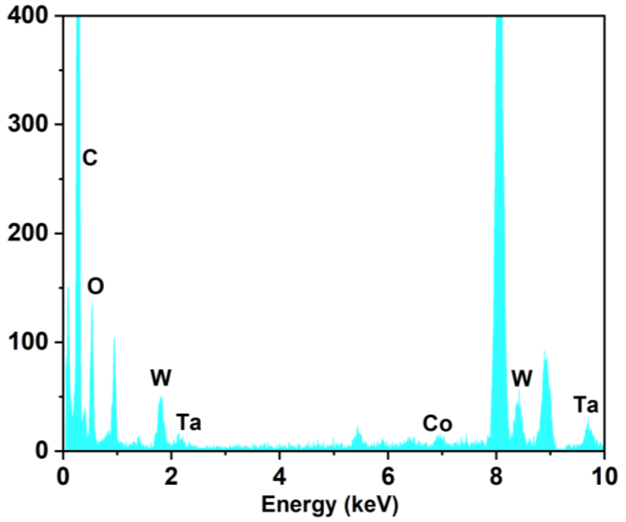

Figure S7. EDX the elemental mapping of C, Co, W and Ta.

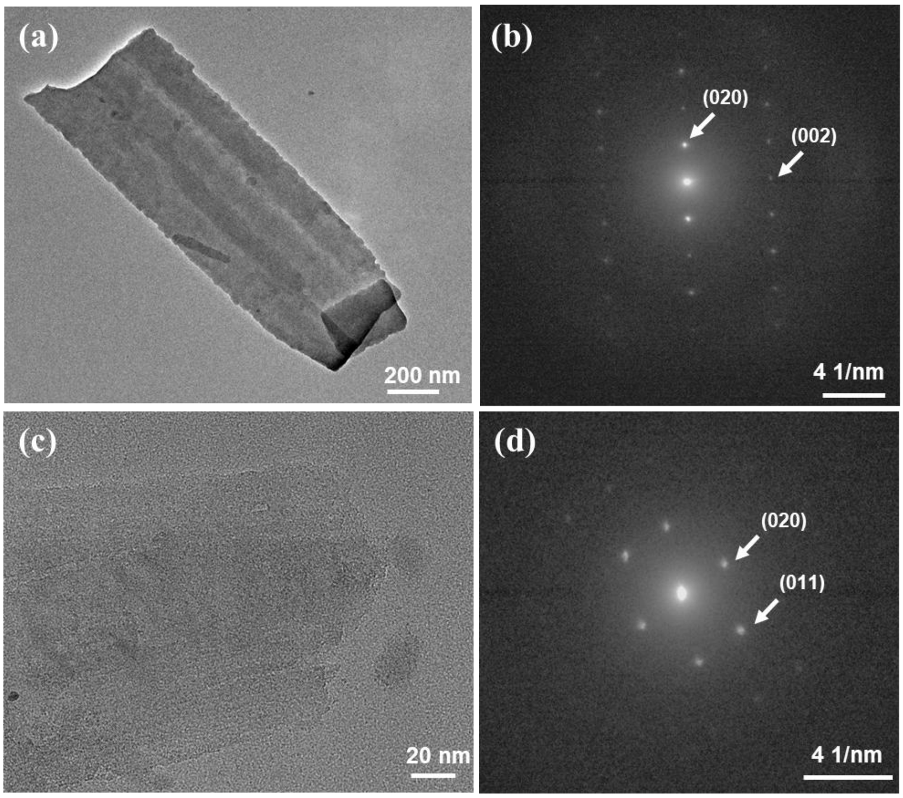

Figure S8. (a) TEM image of the **Bi-NS-1**. (b) Selected area electron diffraction (SAED) pattern of the **Bi-NS-1**, showing good agreement with the FFT pattern and simulated electron diffraction (ED) pattern (Figure 2c, S4b). (c) TEM image of the **Mono-NS-1**. (d) Selected area electron diffraction (SAED) pattern of the **Mono-NS-1**, showing good agreement with the FFT pattern and simulated electron diffraction (ED) pattern (Figure 2g, S4d).

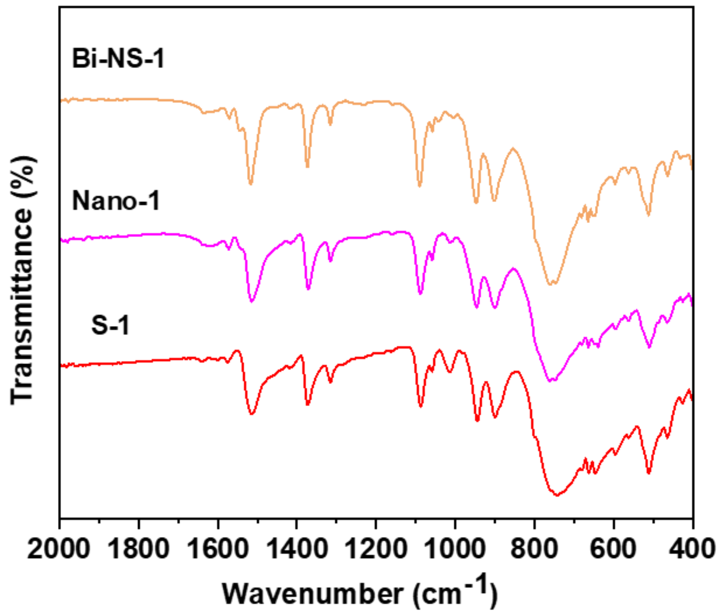

Figure S9. FT-IR spectra of **S-1**, **Nano-1** and **Bi-NS-1**

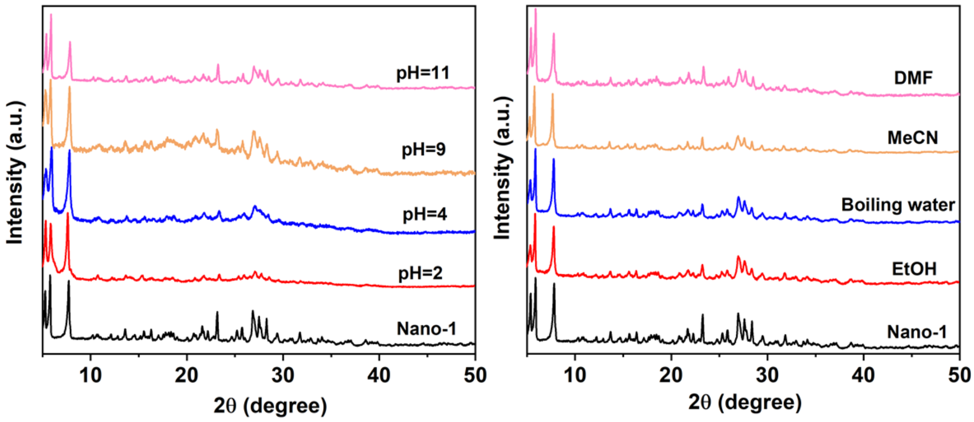

Figure S10. PXRD patterns of **Nano-1** after being immersed in aqueous solution with different pH and DMF, MeCN, boiling water, and EtOH for 12h.

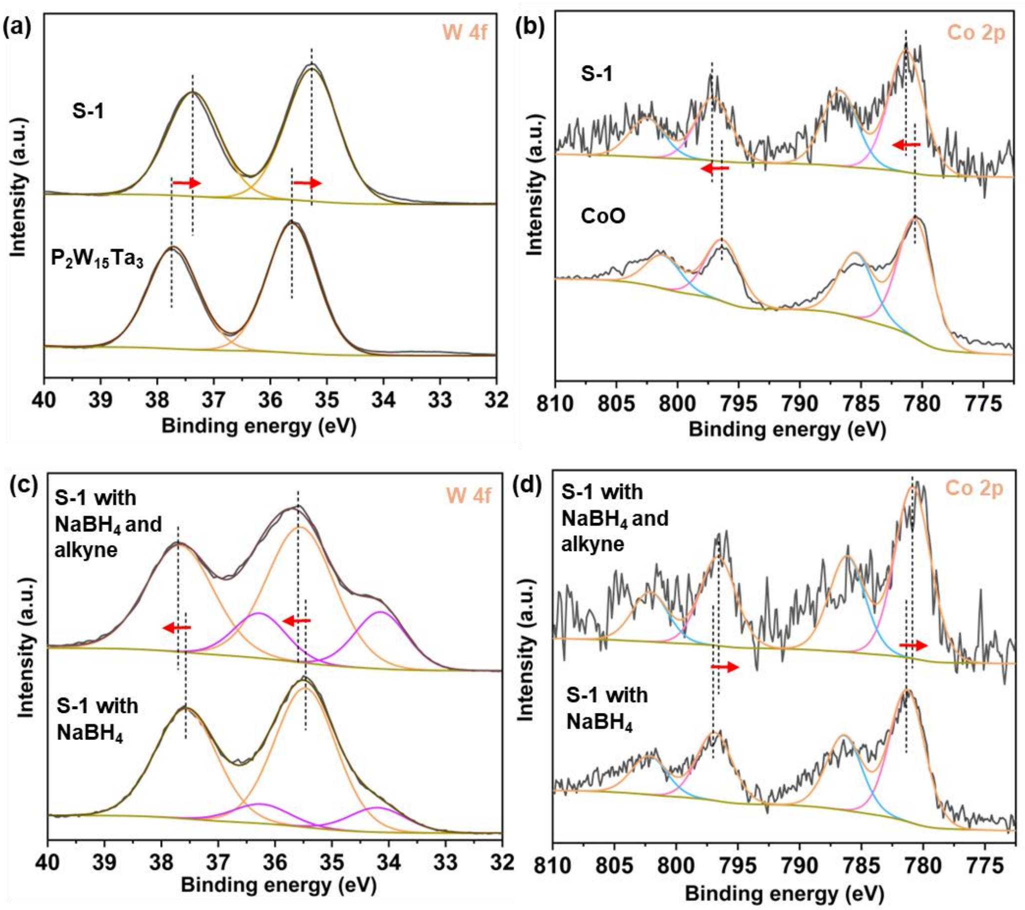

Figure S11. (a) W 4f binding energy comparison between {$P_2W_{15}Ta_3$} and pristine **S-1**. (b) Co 2p binding energy comparison between pristine **S-1** and CoO. (c) W 4f binding energy comparison of **S-1** after treatment with $NaBH_4$ and upon subsequent alkyne addition. (d) Co 2p binding energy comparison of **S-1** after treatment with $NaBH_4$ and upon subsequent alkyne addition. The arrows point in the direction of binding energy shift. Sample treatment procedure for (c) and (d): all manipulations were carried out in a glovebox. In a reaction vial equipped with a magnetic stir bar, the catalyst (10 mg) and sodium borohydride (0.5 mmol) were combined. Methanol (3 mL) was then added, and the mixture was stirred for 1 min. A portion of the catalyst was separated and subjected to XPS analysis. Subsequently, 0.2 mmol of the alkyne was added to the remaining catalyst. After 1 min of reaction, the catalyst was isolated and analyzed by XPS.[40]

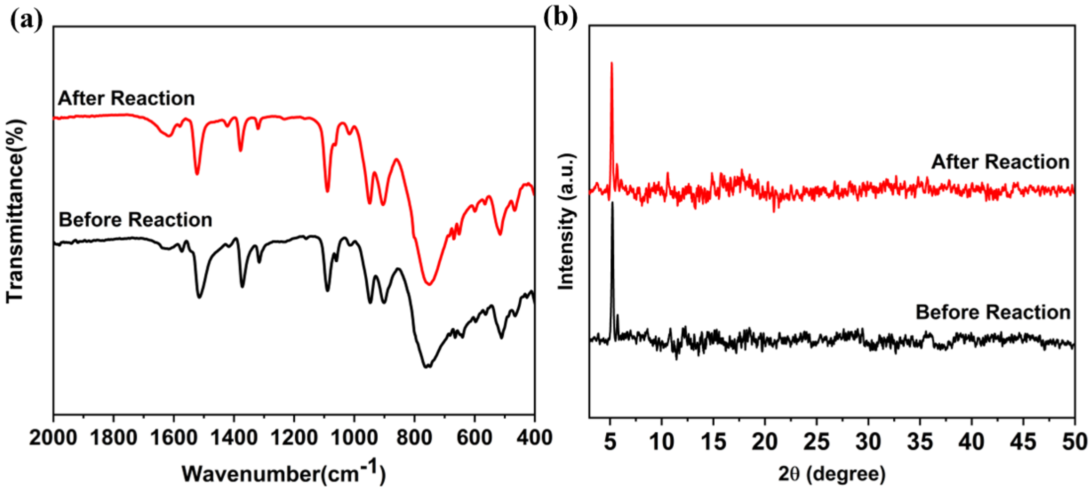

Figure S12. The IR spectra (a) and Powder XRD patterns, (b) of **Bi-NS-1** before and after catalysis.

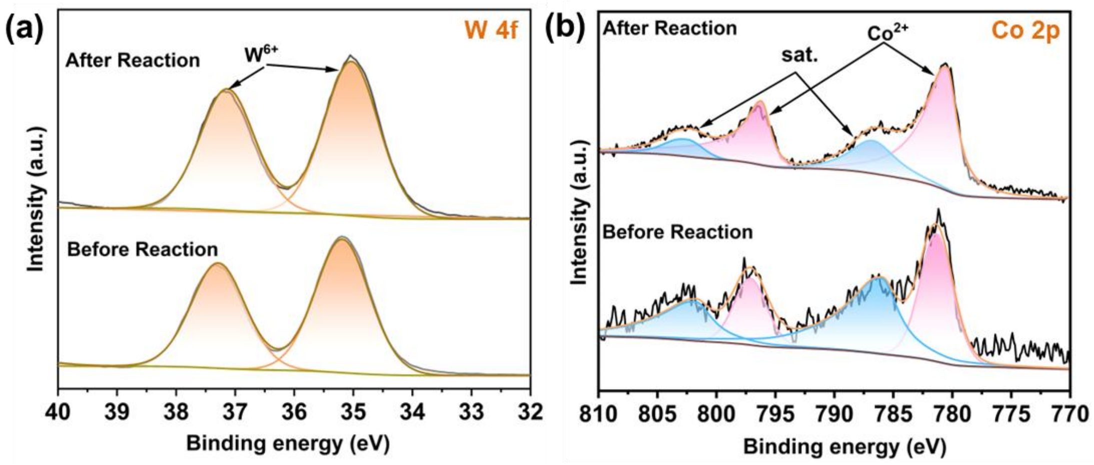

Figure S13. W 4f and Co 2p XPS spectra of **Bi-NS-1** before and after catalysis (The catalyst after reaction was washed three times using methanol, then placed in the air for 1 hour to return to orange-yellow and then subjected to XPS testing).

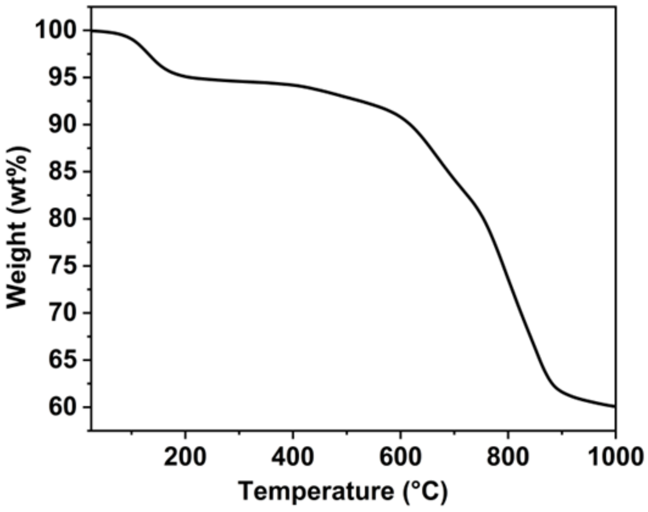

Figure S14. The TGA curve of **S-1** measured in N_2 from 25 °C to 1000 °C.

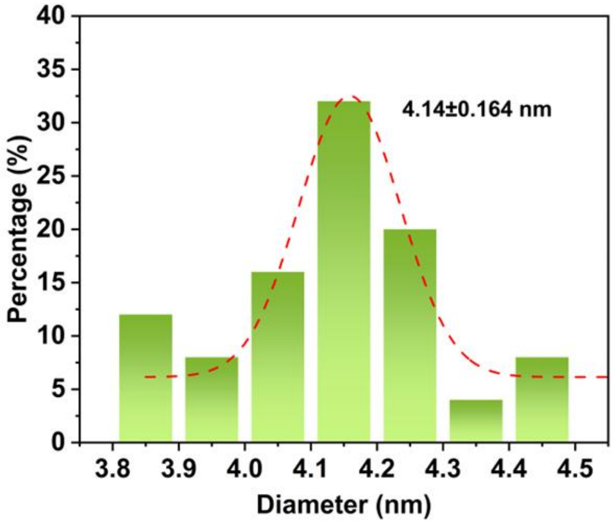

Figure S15. The statistical distribution of AFM thickness for **Bi-NS-1**.

S5 Catalytic Alkyne Semi-Hydrogenation

Alkyne semi-hydrogenation: The catalyst was vacuum-dried at 80 °C for 1 h prior to use. The respective catalysts (1.6 μmol, 10 mg), the substrate (0.2 mmol) and $NaBH_4$ (0.5 mmol, 19 mg) were placed into a reaction tube containing a magnetic stir bar. The tube was sealed with a silicone septum, evacuated and backfilled with argon (three cycles). After the final cycle, the pressure was adjusted to slightly below atmospheric pressure, and then MeOH (3 mL) was injected under reduced pressure. The reaction mixture was stirred at room temperature (r.t.). Upon completion, the catalyst was rapidly removed by filtration through a syringe filter. The yield of the semi-hydrogenation products was determined by gas chromatography-mass spectrometry (GC-MS) with naphthalene as an internal standard. The catalyst was recovered by centrifugation and washing with MeOH for subsequent use.

Table S4. Substrate scope for the semi-hydrogenation of internal alkynes catalysed by **Bi-NS-1.**

Entry	Substrate	Conv. (%)	Alkene Sel. (%)	Z:E
1	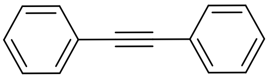	99	100	85:15
2	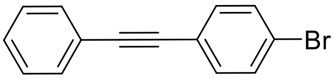	99	100	86:14
3	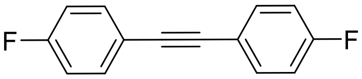	99	100	85:15
4	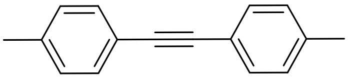	99	100	83:17
5	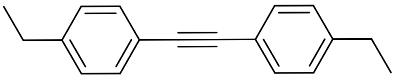	99	100	82:18
6	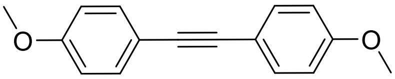	99	100	99:1

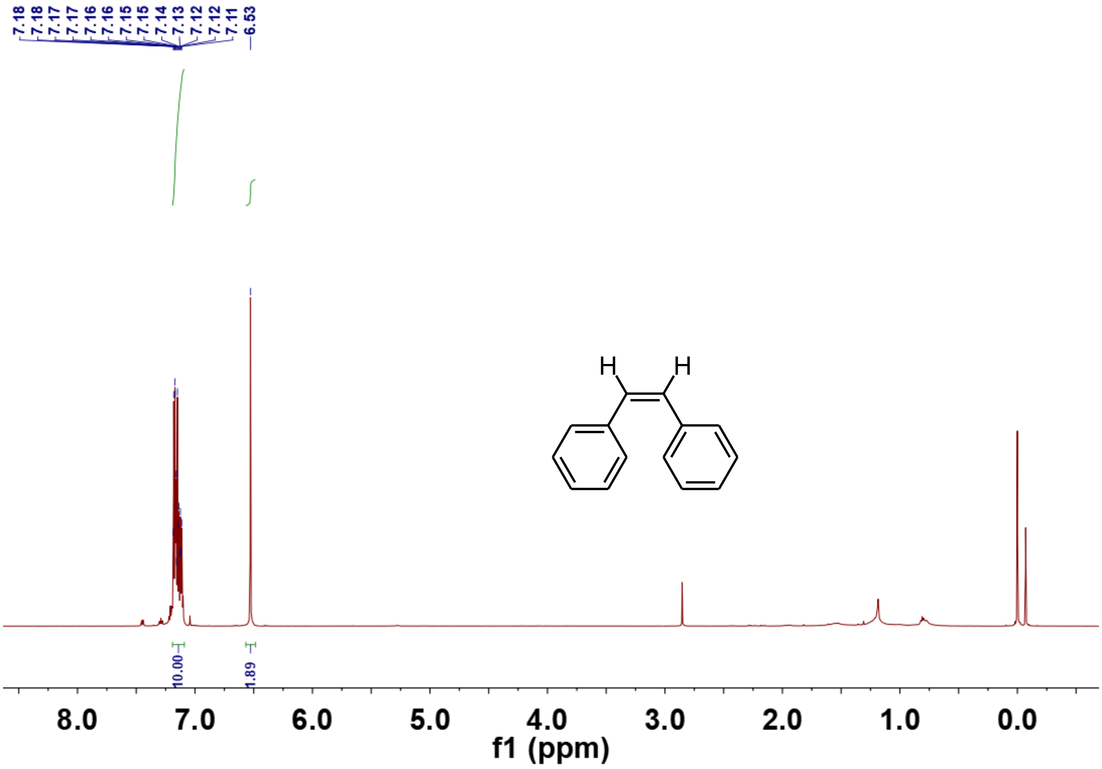

Figure S16. ^{1}H NMR of stilbene using $NaBH_4$ and CH_3OH in $CDCl_3$.

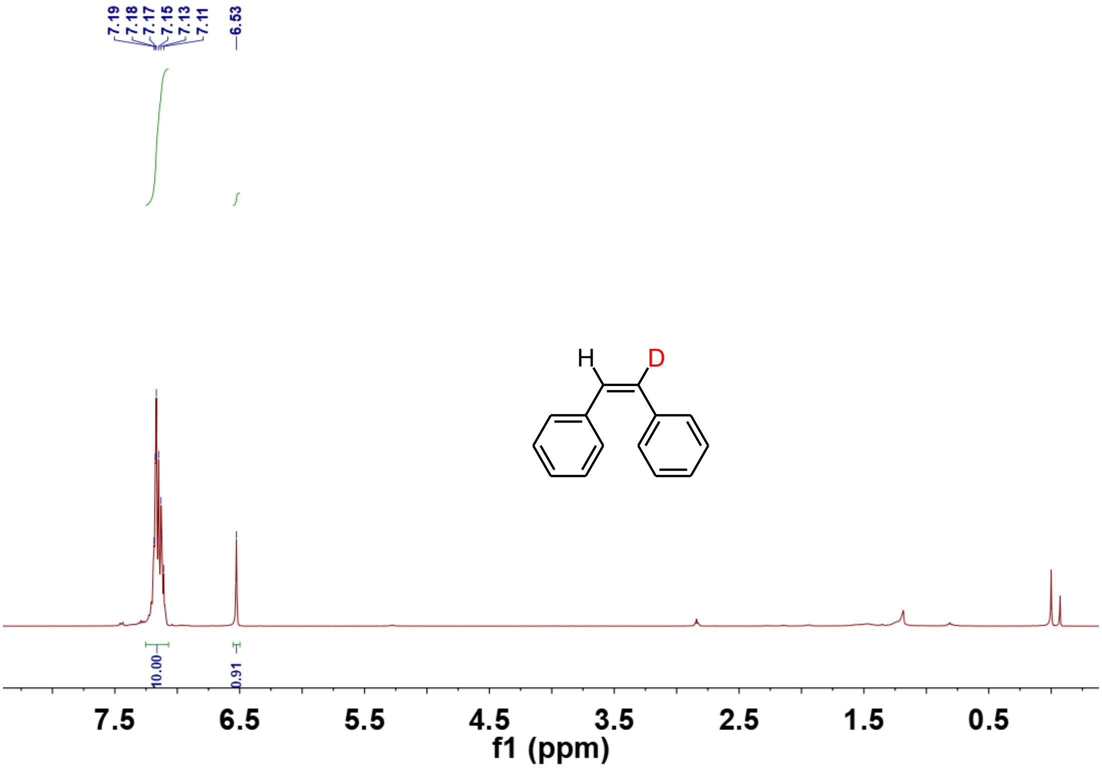

Figure S17. ^{1}H NMR of monodeuterated stilbene using $NaBD_4$ and CH_3OH in $CDCl_3$.

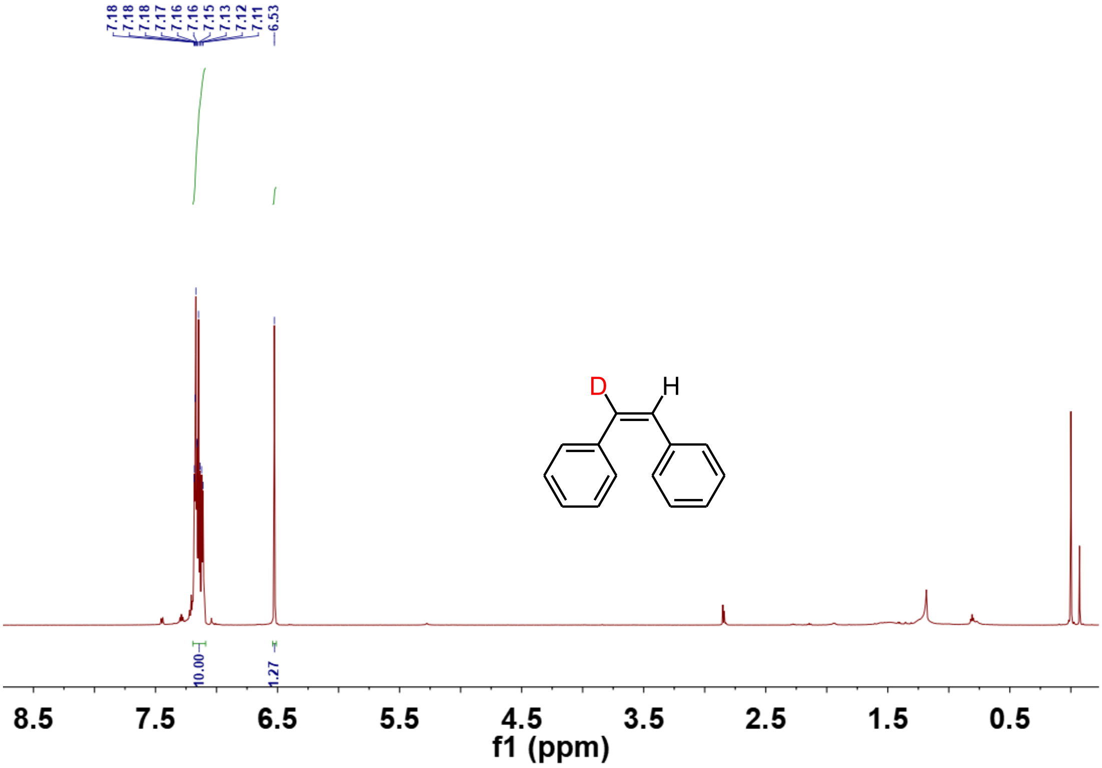

Figure S18. ^{1}H NMR of monodeuterated stilbene using $NaBH_4$ and CH_3OD in $CDCl_3$.

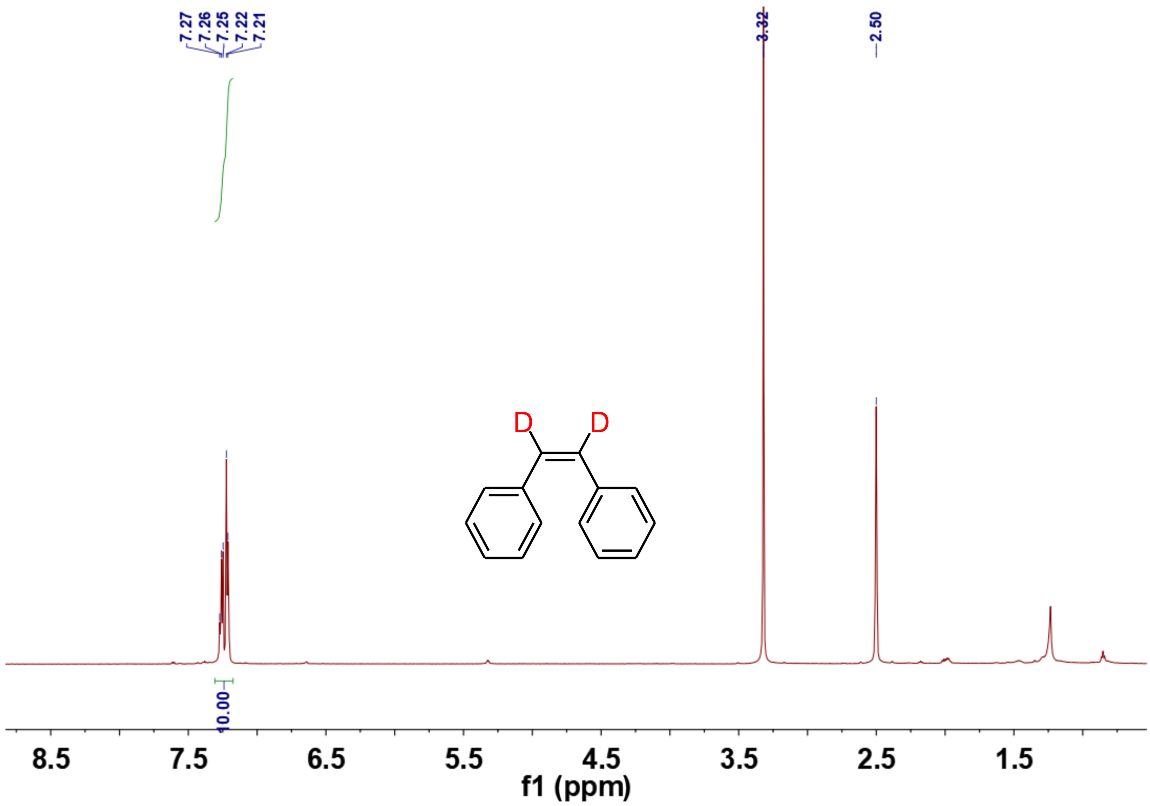

Figure S19. ^{1}H NMR of dideuterated stilbene using $NaBD_4$ and CH_3OD in DMSO-d_6.

Table S5. A summary of $NaBH_4$ as a reducing agent for the reduction of alkynes.

Catalyst Type	Specific Catalyst Composition	Alkyne Substrate	Time	Conversion	Selectivity (Product Ratio)	Ref.
Pd-based supported	$Pd(OAc)_2$ (Pd nanoparticles formed via $NaBH_4$ reduction)	Alkyne	1 h	100%	Z-alkenes	[41]
Ni-based complexes	[(dippe)Ni(η²-C,C-dpa)] (Diphenylacetylene Ni(0) complex)	Diphenylacetylene	72 h	100%	cis-Stilbene:trans-Stilbene = 4:96	[42]
	[(dtbpe)Ni(η²-C,C-dpa)] (Diphenylacetylene Ni(0) complex)	4-Methoxyphenylacetylene	72 h	100%	trans-4-Methoxystyrene (98%), cis-4-Methoxystyrene (2%)	
	$[(dippe)Ni(\mu\text{-}H)]_2$ (Ni(I) hydride dimer, in-situ generated Ni(0))	Phenylacetylene	24 h	97%	Styrene (97%), Ethylbenzene (3%)	
Co-based bimetallic	$CuSO_4/CoCl_2$	Diphenylacetylene	15 min	100%	cis-Stilbene:trans-Stilbene:Bibenzyl = 49:26:25	[43]
Porphinatoiron complexes	$(TPP)Fe^{3+}Cl$ (Tetraphenylporphyrinatoiron(III) chloride)	Phenylacetylene	24 h	17%	Styrene (17%), Ethylbenzene (23%)	[44]
	$(TPP)Fe^{3+}Cl$ (Tetraphenylporphyrinatoiron(III) chloride)	1,2-Diphenylacetylene	1 h	77%	1,2-Diphenylethane (77%), cis-Stilbene (1%), trans-Stilbene (trace)	

S6 References

[37] S. Li, S. Liu, S. Liu, Y. Liu, Q. Tang, Z. Shi, S. Ouyang and J. Ye, *J. Am. Chem. Soc.* **2012**, *134*, 19716-19721.

[38] G. Sheldrick, *Acta Cryst. A* **2008**, *64*, 112-122

[39] O. V. Dolomanov, L. J. Bourhis, R. J. Gildea, J. A. K. Howard, H. Puschmann, *J. Appl. Cryst.* **2009**, *42*, 339-341.

[40] H. Li, Q. Lu, F. Zhang, Q. Liu, J. Zhuang, Z. Li, X. Wang, *Nat Commun.* **2025**, *16*, 5778.

[41] E. D. Slack, C. M. Gabriel, B. H. Lipshutz, *Angew. Chem. Int. Ed.* **2014**, *53*, 14051-14054.

[42] R. Barrios-Francisco, J. García, *Appl. Catal. A.* **2010**, *385*, 108-113.

[43] M. Ficker, S. Svenningsen, T. Larribeau, J. Christensen, *Tetrahedron Lett.* **2018**, *59*, 1125-1129.

[44] M. Takeuchi, K. Kano, *Organometallics* **1993**, *12*, 2059-2064.
